# Disparities in Cutaneous T-Cell Lymphoma Incidence by Race/Ethnicity and Area-Based Socioeconomic Status

**DOI:** 10.3390/ijerph20043578

**Published:** 2023-02-17

**Authors:** Daniel Wiese, Antoinette M. Stroup, Alina Shevchenko, Sylvia Hsu, Kevin A. Henry

**Affiliations:** 1 Department of Surveillance and Health Equity Science, American Cancer Society, Kennesaw, GA 30144, USA; 2 Department of Geography and Urban Studies, Temple University, Philadelphia, PA 19122, USA; 3 New Jersey State Cancer Registry, New Jersey Department of Health, Trenton, NJ 08608, USA; 4 Rutgers Cancer Institute of New Jersey, Rutgers Biomedical and Health Sciences, New Brunswick, NJ 08901, USA; 5 Department of Biostatistics and Epidemiology, Rutgers School of Public Health, Piscataway, NJ 08854, USA; 6 Department of Dermatology, Lewis Katz School of Medicine at Temple University, Philadelphia, PA 19140, USA; 7 Division of Cancer Prevention and Control, Fox Chase Cancer Center, Philadelphia, PA 19115, USA

**Keywords:** cutaneous T-cell lymphoma, disease mapping, spatial analysis, income disparities, racial disparities

## Abstract

Cutaneous T-cell lymphoma (CTCL) is a rare type of extranodal non-Hodgkin lymphoma (NHL). This study uses population-based data from the New Jersey (NJ) State Cancer Registry to examine geographic variation in CTCL incidence and evaluates whether CTCL risk varies by race/ethnicity and census tract socioeconomic status (SES). The study included 1163 cases diagnosed in NJ between 2006 and 2014. Geographic variation and possible clustering of high CTCL rates were assessed using Bayesian geo-additive models. The associations between CTCL risk and race/ethnicity and census tract SES, measured as median household income, were examined using Poisson regression. CTCL incidence varied across NJ, but there were no statistically significant geographic clusters. After adjustment for age, sex, and race/ethnicity, the relative risk (RR) of CTCL was significantly higher (RR = 1.47, 95% confidence interval: 1.22–1.78) in the highest income quartile than in the lowest. The interactions between race/ethnicity and SES indicated that the income gradients by RR were evident in all groups. Compared to non-Hispanic White individuals in low-income tracts, CTCL risk was higher among non-Hispanic White individuals in high-income tracts and among non-Hispanic Black individuals in tracts of all income levels. Our findings suggest racial disparities and a strong socioeconomic gradient with higher CTCL risk among cases living in census tracts with higher income compared to those living in lower-income tracts.

## 1. Introduction

Cutaneous T-cell lymphoma (CTCL) is a rare type of extranodal non-Hodgkin lymphoma (NHL), which includes multiple subtypes, such as mycosis fungoides and Sézary syndrome [1,2]. CTCL primarily affects the skin and accounts for approximately 4% of all NHL cases. In the United States, according to Surveillance, Epidemiology, and End Results (SEER)*Stat database [3], the incidence rate of CTCL is about 12.4 cases per million persons per year. Differences in the incidence rates by sex, age, and race have been previously reported. Men have higher rates than women, and CTCL incidence is slightly higher in Black people compared to all other race groups [3]. The risk of CTCL increases with age, with an average onset between 50 and 60 years [4]. Typically, CTCL develops with abnormalities in T-cells, which attack the skin, and may progress from erythematous, scaly, round patches to plaques and occasionally tumors [5].

There are several challenges when studying CTCL. First, the cause of CTCL remains unknown. Case reports and studies have suggested that infectious agents could play a role in the development of CTCL in couples [6,7,8] or genetically related relatives [9,10,11]. Additionally, numerous occupational risk factors, including chemical exposures from employment in paper, glass, pottery, or ceramics manufacturing industries [12], occupational exposures to petrochemicals [13,14], halogenated hydrocarbons [15], or pesticides [16], and chronic occupational exposure to solar radiation [17] are reported to be associated with increased risk of CTCL. However, individual-level factors, such as a family history of multiple myeloma, a history of eczema, a history of T-cell activating autoimmune conditions, a BMI of 30+ kg/m2, as well as behavioral factors, such as a long duration of cigarette smoking and low physical activity were associated with increased risk [18]. Studies have also noted that patients with HIV-related immunodeficiency [19], as well as organ transplant recipients, had an increased risk of developing CTCL [20].

A few studies examining geographic variation in CTCL incidence rates across the United States and Canada have reported regional variations in incidence rates and clustering [21,22,23,24,25,26,27]. Several of these studies found higher rates in industrial and urban regions and suggest a potential association between CTCL incidence and environmental exposure [25,26,28,29]. Another study from Pittsburgh, Pennsylvania, however, also suggests that the clustering of higher CTCL incidence may be related to the density of dermatologists, which increases the likelihood of definitive diagnosis [24].

The main objectives of this cross-sectional study were to examine the geographic distribution and potential clustering of CTCL incidence in New Jersey (NJ) based on the residence at the time of diagnosis and to assess whether the risk of CTCL varies by race/ethnicity and census tract socioeconomic status. To our knowledge, this is one of the first geographic studies to use Bayesian geospatial models to assess the potential geographic clustering of CTCL and map the geographic variation of CTCL incidence using population-based data. 

## 2. Materials and Methods

### 2.1. Study Population

The study population included 1163 NJ residents, 18 years and older, diagnosed with histologically confirmed, first primary, invasive CTCL from 2006 to 2014. Cases were ascertained from the NJ State Cancer Registry (NJSCR) and defined [based on International Classification of Diseases for Oncology, 3rd Edition (ICD-O-3)] histologies including: 9700, mycosis fungoides; 9701, Sézary syndrome; 9702, 9705, 9708 and 9714, 9718, 9719, 9827; 9709 and 9726, NOS CTCL cases). Information about patient demographic characteristics, including date of diagnosis, sex, race, and ethnicity, was also provided by the NJSCR. Race/ethnicity was classified into Non-Hispanic White (NHW), Non-Hispanic Black (NHB), Asian/Pacific Islanders (API), and Other race. Residential address from the time of diagnosis was geocoded by the NJSCR, using the North American Association of Central Cancer Registries (NAACCR) Automated Geospatial Geocoding Interface Environment (AGGIE) Geocoder [30] and linked to 2010 census tracts.

### 2.2. Socioeconomic Data

To estimate area-based socioeconomic status (SES), we used census tract (CT) median annual household income (“income”) and the percent of the adult population living below the federal poverty line (“poverty”). SES data were obtained from the social explorer, using American Community Survey 5-year averages. All income values were adjusted to the 2019 US dollar value and transformed into quartiles. Poverty data were also categorized into quartiles. A quartile value was assigned to each CTCL case based on residential CT at the time of diagnosis.

### 2.3. Population Data

Population counts for the years 2006–2014 by age, sex, and race/ethnicity for census tracts used in subsequent analyses were obtained from the National Cancer Institute (NCI) Surveillance, Epidemiology, and End Results (SEER) [3]. Because population estimates for CTs were not available for the other race group, these cases were excluded (*n* = 79) from the further analysis.

### 2.4. Statewide Incidence

The statewide incidence rate of CTCL was estimated using SAS v. 9.3 (SAS Institute, Inc., Cary, NC, USA) as an average annual number of CTCL cases per 100,000 population. All of the rates were age-standardized to the 2000 US Census population (19 age groups) by means of the direct method. The 95% confidence intervals (CI) for incidence rates were calculated as gamma intervals. All tests of statistical significance and confidence intervals were two-sided. A *p*-value less than 0.05 was considered statistically significant.

### 2.5. Geographic Modeling

Geographic modeling and cluster detection analysis is a widely applied technique that can be implemented in cross-sectional studies to evaluate whether the number of cancer cases is greater than expected within a group of people, a geographic area, or a period of time [31]. To estimate the variation in incidence rates across the study area, Poisson regression is a widely accepted model available in several geospatial statistical packages. 

The coefficients for each covariate (fixed effect) and the geographic standardized incidence ratios (SIR) of CTCL by CT were estimated using a geospatial Poisson regression model adjusting for sex, age at diagnosis, race/ethnicity, and the spatial effect based on the residential CT at the time of diagnosis, using the following equation:

(1) $$Oi\sim Poisson\left(Ei\;\lambda_{i}\right)$$

where Oi and Ei are the observed and expected numbers of cases, respectively, in area i, and λi refers to the SIR in each CT.

(2) $$\log\left(\lambda_{i}\right)=\eta_{i}=b_{0}+offset\left(\log\left(population\right)\right)+f_{1}\left(x_{i1}\right)\ldots +f_{k}\left(x_{ik}\right)+f_{spat}^{1}\left(s_{i}\right)+U\left(s_{i}\right)$$

$\eta_{i}$ refers to the number of CTCL cases in each CT (i) aggregated by sex, age group, and race/ethnicity. The model intercept, $b_{0}$, is the statewide average SIR across all CTs in NJ, while the offset is the natural logarithm of the CT-population aggregated by sex, age group, and race/ethnicity. Additionally, the model includes $f_{1}\left(x_{i1}\right)\ldots +f_{k}\left(x_{ik}\right),\;$representing a function for covariates 1 to k (e.g., income and poverty), and the structured spatial effect $f_{spat}^{1}\left(s_{i}\right)$ [32], defined as a stationary Gaussian random field specification based on the neighboring CTs adjacency matrix (rook’s case) [33].

The Poisson model was implemented using Bayesian inference using Markov Chain Monte Carlo (MCMC) simulation. For each model, we ran 10,000 iterations, with the first 2000 samples used as a burn-in. From the remaining 8000 samples, every 10th was used to construct the posterior distribution for each of the parameter estimates (non-spatial and spatial) in the model. All of the models were implemented with R statistical software, using BayesX [32], BayesXsrc [34], and R2BayesX [35].

We first developed several models adjusted for each CT SES variable at a time (categorized into quartiles) to estimate the crude coefficients. We then adjusted all models for age at diagnosis. Finally, we ran a full multivariable model, adjusted for age at diagnosis, sex, race/ethnicity, spatial effect, and the CT measure of SES. Covariate coefficients were exponentiated and interpreted as relative risks (RR). The CTCL risk by income and poverty in each of the 3 highest quartiles (Q2–Q4) were compared to the lowest quartile (Q1). We also tested for joint effects of race/ethnicity and income on CTCL incidence by inserting interactive terms in the model, using NHW in the highest income quartile as the reference group. The spatial coefficients for each CT were exponentiated as the SIR and mapped using QGIS 3.10.

All of the models were compared using the statistical measure of model fit (Deviance Information Criterion, DIC), SIR Range, and the percentage of unexplained geographic disparity (GD). A lower value of DIC suggests a better model fit [36]. The attenuation of the SIR range closer to 1 after adding each new covariate suggests a more robust explanatory effect of the variation in SIR, and lower GD values suggest a greater impact in explaining remaining geographic variability (i.e., disparities).

## 3. Results

The direct age-standardized incidence rate was 1.37 cases per 100,000 persons. CTCL incidence rates increased with age and were highest among 70–79 year-olds (Table 1).

The age-adjusted incidence rates were statistically significantly higher for men compared to women (*p* < 0.05) (Table 2). The age-adjusted incidence rates by race/ethnicity were highest in NHB (aaIR 1.63 95% CI 1.39–1.91). The second highest incidence rates were for NHW (aaIR 1.24, 95% CI 1.39–1.91). Age-adjusted incidence rates were highest in the uppermost SES quartile for income (aaIR 1.51 95% CI 1.33–1.70) and poverty (aaIR 1.37 95% CI 1.20–1.54) (Table 2).

The results from the fixed-effects geospatial Poisson regression models indicated that CTCL risk was significantly lower for women compared to men and significantly higher in NHB compared to NHW after adjusting for age and sex (Table 3). Across all models, the risk of CTCL was significantly higher in the uppermost income quartile compared with the lowest. The multivariable-adjusted model showed that the risk of CTCL was significantly higher among cases in the highest income quartile after adjustment for individual-level factors (RR 1.47 95% CI 1.22–1.78). When examining the RR of CTCL by CT poverty, we observed similar directionality and significance: the risk of CTCL was highest in the wealthiest group (RR 1.31 95% CI 1.08–1.58).

**Figure 1:**
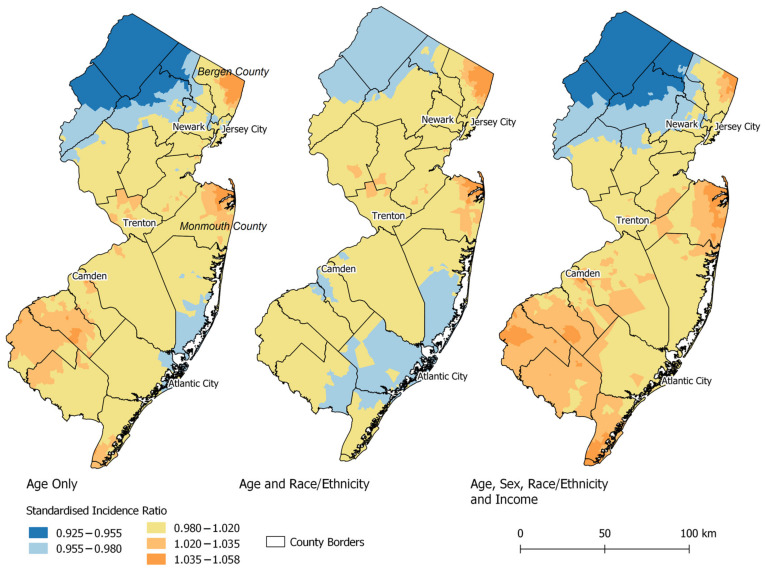
Geographic risk estimates (Standardized Incidence Ratio) of CTCL across New Jersey.

The geographic risk of CTCL estimates varied across NJ. The DIC was lowest in the full individual-level multivariable model (16,487.0). The SIR range was highest in the sex-only model (0.85–1.18) and lowest in the age-only model adjusted (0.96–1.04). The full individual-level multivariable model had an SIR range of 0.93–1.07. Comparing the percentage of unexplained spatial variance, the GD was 10.35% in the age-adjusted model, 12.29% when adjusting for age, sex, and race/ethnicity, and 12.57% after adjustment for all individual-level variables and CT median income. When evaluating CT poverty, the GD increased to 15.94%, indicating a larger proportion of unexplained geographic disparities (Supplemental Table S1). There were no statistically significant areas of elevated CTCL risk in NJ in comparison to the statewide average in any model (Figure 1). Generally, the SIR of CTCL was below 1.0 (lower risk than the average statewide rate) in northern NJ and above 1.0 (higher risk than the average statewide rate) in parts of Bergen and Monmouth counties.

Table 4 describes the joint effects of race/ethnicity and CT median household income on CTCL RR, using NHW in the lowest CT median household income areas (Q1) as the reference group. After controlling for age, sex, and spatial effect, NHBs in all income categories had a significantly higher RR of CTCL. NHWs in the highest CT median household income group had a significantly higher risk of CTCL compared to their less-affluent counterparts (Q4 RR 1.39 95% CI 1.1–1.74). Income gradients were also evident in all race/ethnic groups, but there were no significant differences in RR among Hispanics and API. When using CT poverty, a higher risk of CTCL was significant for NHW and NHB from areas with very low and low poverty levels as well as for high-poverty area NHB (Supplemental Table S2).

## 4. Discussion

We did not detect any areas in NJ where the relative risk of CTCL was statistically significantly higher than the statewide average. The smoothed SIR maps showed only minor regional differences in CTCL risk statewide. The results from the regression models indicated a statistically significant association between CT median household income and CTCL risk in NJ, independent of age, sex, and race/ethnicity. We also uncovered interesting race/ethnicity and SES interactions, providing additional evidence of the combined racial and income disparities of CTCL risk. These results were also consistent when using CT poverty as an SES measure.

In contrast to several studies in other regions that found geographic clustering of CTCL [21,24,25,26], there was no evidence of clustering in NJ. There are several possible reasons why we did not find any evidence of geographic clustering. The location of diagnosis might not be a good indicator when trying to detect clusters because of residential mobility. While the exact cause of CTCL is unknown, if there are, in fact, geographic or occupational exposures related to CTCL risk, the exposure might have occurred at previous residential locations. For CTCL, geographic mobility could be important because of the known delays in diagnosis [37]. CTCL often mimics more common skin conditions, such as eczema and psoriasis, and because of general difficulty in accurately diagnosing patients, numerous studies have reported diagnostic delays lasting from a few months to a few years between first symptom development and initial CTCL diagnosis [37,38,39]. 

A second reason why we did not find significant geographic clustering of CTCL may be related to the geography of NJ, which impacts access to a dermatologist. In a previous study from Pittsburgh, the authors noted that the cluster of CTCL was attributed to the higher density of dermatologists in the area [24]. NJ is a highly densely populated state with short travel times and good geographic access to dermatologists statewide. Therefore, these factors might be contributing to a more even distribution of CTCL statewide. The slightly elevated SIRs in some NJ counties could be related to two factors: the high income of the counties (e.g., Bergen, Monmouth) or their close proximity to major healthcare providers in NJ, New York City, and Philadelphia.

We also found that people living in areas of high income had a higher risk of CTCL than those living in lower-income areas. The underlying factors that may account for this association, however, are not known, but there might be a possible explanation: having higher income and potentially greater access to health care would allow more frequent dermatologist visits, which may result in a timely diagnosis. In contrast, populations from lower-income areas may not afford regular skin checks and may die of CTCL or some other disease while not being diagnosed. It is conceivable that area-based median household income is correlated with behavioral risk factors that modify the risk of CTCL. It is also possible that differences in residential location, based on an individual’s income, could result in variations in exposure to environmental or occupational risk factors that are hypothesized to be associated with CTCL.

Our study also revealed important associations between increased CTCL incidence rates and interactions between patients’ race/ethnicity and SES. Compared to NHW people in the least affluent areas, the most affluent NHW and NHB people of any SES had a higher risk of CTCL. CTCL risk among NHB people in the most affluent areas reached significance at 88% higher than their NHW counterparts. We also found suggestive evidence of a consistent SES gradient among all race/ethnic groups. This could have two different potential explanations: for NHB people, higher rates of CTCL could be related to a higher prevalence of more severe eczema or atopic dermatitis [40,41,42], potentially attributed to genetic susceptibility [42,43]. For high SES patients, higher rates could be the result of a greater frequency of diagnosis. A higher prevalence of atopic dermatitis is more common in wealthy and educated populations [44]. In contrast, severe eczema is reported to be more common in low SES patients [42], which are a relatively large group among NHB patients (*n* = 86; 52%) than among NHW patients (*n* = 111; 15%) in this study.

This study has several limitations. First, this study utilized only residential locations at the time of diagnosis. This limits our ability to examine the geographic clustering of incidence based on previous exposures. It also limits our ability to assess area-based SES longitudinally over the life course. Second, we did not have access to individual SES as this is not routinely collected on a population-based level by cancer registries. Third, given the difficulty in diagnosing CTCL, there may be undiagnosed CTCL cases. If undiagnosed cases are highest among people living in lower-income areas, this could be a source of differential misclassification and an underestimate of CTCL rates in that population. Fourth, the relatively low number of cases could result in a reduced power to detect significant geographic clustering. Having more years of CTCL cases could potentially result in the identification of areas with significantly higher CTCL incidence rates.

## 5. Conclusions

The exact causes of CTCL remain largely unknown. Our findings suggest a strong socioeconomic gradient and racial disparities with the increasing risk being associated with higher SES and the highest CTCL rates in affluent NHB people. However, we did not detect any statistically significant geographic clustering of CTCL incidence in NJ. Future studies should examine the geographic variations of CTCL in other states and include information about individual-level SES and health insurance.

## Figures and Tables

**Table 1:** Age-specific crude incidence rates of CTCL cases diagnosed from 2006 to 2014 in New Jersey, *n* = 1084.

Age at Diagnosis, Years	*n*	%	Crude IR *
<30	60	5.5	1.22
30–39	94	8.7	1.80
40–49	153	14.1	2.53
50–59	232	21.4	4.23
60–69	242	22.3	6.62
70–79	197	18.2	8.99
≥80	106	9.8	6.64

IR: Incidence Rate. * Per 100,000 person-years.

**Table 2:** Age-adjusted incidence rates by sex, race, ethnicity, and assigned census tract median household income and poverty categories for CTCL cases diagnosed from 2006 to 2014 in New Jersey, *n* = 1084.

Characteristic	n	%	Age-Adjusted IR *^†‡^	95% CI
**Sex**				
Female	631	58.21	0.98	0.89–1.08
Male	453	41.79	1.58	1.46–1.71
**Race/Ethnicity**				
Non-Hispanic White	733	67.62	1.24	1.14–1.33
Non-Hispanic Black	166	15.31	1.63	1.39–1.91
Hispanic	125	11.53	1.14	0.94–1.37
Asian/Pacific Islander	60	5.54	0.91	0.68–1.18
**CT Median Income**				
Very High (≥$109,665)	271	25.0	1.51	1.33–1.70
High ($80,382–109,665)	270	25.0	1.17	1.05–1.33
Medium ($58,927–80,382)	271	25.0	1.25	1.10–1.41
Low (<$58,927)	272	25.0	1.17	1.04–1.32
**CT Poverty**				
Very Low (<2.89%)	270	25.0	1.37	1.20–1.54
Low (2.89–5.48%)	271	25.0	1.26	1.11–1.43
Medium (5.48–10.34%)	271	25.0	1.23	1.09–1.39
High (10.34%+)	272	25.0	1.17	1.03–1.32

Note: IR: Incidence Rate; CI: Confidence Interval; CT: Census tract. * Per 100,000 person-years; age-adjustment to the 2000 US Standard Population (19 age groups). ^†^ Denominators for incidence rates were based on 2010 U.S. census populations. ^‡^ Directly age-adjusted rate.

**Table 3:** Relative risk of CTCL by sex, race/ethnicity, census tract median household income and poverty for cases diagnosed in 2006–2014 in New Jersey *n* = 1084 *.

Characteristic	Crude RR (95% CI)	Age-Adjusted RR (95% CI) ^†^	Multivariable-Adjusted RR (95% CI) ^‡^
**Sex**			
Male	Referent	Referent	Referent
Female	0.68 (0.61–0.76)	0.59 (0.52–0.66)	0.59 (0.52–0.67)
**Race/Ethnicity**			
Non-Hispanic White	Referent	Referent	Referent
Non-Hispanic Black	1.02 (0.86–1.2)	1.39 (1.17–1.63)	1.56 (1.29–1.89)
Hispanic, Any Race	0.57 (0.47–0.69)	0.94 (0.76–1.17)	1.06 (0.86–1.29)
Asian/Pacific Islander	0.56 (0.43–0.72)	0.8 (0.61–1.04)	0.8 (0.6–1.04)
**CT Median Income**			
Very High (≥$109,665)	1.39 (1.17–1.67)	1.30 (1.10–1.55)	1.47 (1.22–1.78)
High ($80,382–109,665)	1.12 (0.95–1.34)	1.03 (0.86–1.24)	1.14 (0.96–1.38)
Medium ($58,927–80,382)	1.17 (0.98–1.4)	1.10 (0.93–1.32)	1.19 (1.00–1.43)
Low (<$58,927)	Referent	Referent	Referent
**CT Poverty**			
Very Low (<2.89%)	1.38 (1.15–1.64)	1.18 (1.00–1.40)	1.31 (1.08–1.58)
Low (2.89–5.48%)	1.29 (1.09–1.54)	1.12 (0.94–1.33)	1.23 (1.01–1.47)
Medium (5.48–10.34%)	1.21 (1.02–1.43)	1.07 (0.91–1.26)	1.16 (0.97–1.37)
High (10.34%+)	Referent	Referent	Referent

Note: RR: Relative Risk; CT: census tract; CI: 95% Confidence Interval. ^†^ Relative Risk estimates for sex, race, and CT median income and poverty are based on four separate Poisson regression models adjusted for age at diagnosis and spatial effect. ^‡^ Multivariate Poisson regression model adjusted for age, sex, race, CT median income or poverty and spatial effect. * Seventy-nine cases with a race coded “Other” race were excluded from the analysis.

**Table 4:** Distribution and Relative Risk by Race/Ethnicity and CT Median Income Quartile (*n* = 1084 *).

	CT Median Household Income Quartile							
Race/Ethnicity	Very High (≥$109,665)		High ($80,382–109,665)		Medium ($58,927–80,382)		Low (<$58,927)	
	*n* (%)	RR ^‡^ (95% CI)	*n* (%)	RR ^‡^ (95% CI)	*n* (%)	RR ^‡^ (95% CI)	*n* (%)	RR ^‡^ (95% CI)
**Non-Hispanic White**	221 (30)	**1.39 (1.1–1.74)**	216 (29)	1.11 (0.88–1.4)	185 (25)	1.12 (0.88–1.42)	111 (15)	Referent
**Non-Hispanic Black**	18 (11)	**2.61 (1.58–4.29)**	23 (16)	**1.57 (1–2.46)**	39 (22)	**1.76** **(1.22–2.54)**	86 (52)	**1.4 (1.06–1.86)**
**Hispanic**	9 (7)	1.11 (0.56–2.2)	13 (10)	0.83 (0.47–1.47)	35 (28)	1.33 (0.91–1.95)	68 (54)	1.01 (0.75–1.38)
**Asian/Pacific Islander**	23 (38)	1.16 (0.74–1.82)	18 (30)	0.85 (0.51–1.4)	12 (20)	0.75 (0.42–1.37)	7 (12)	0.61 (0.28–1.3)

RR: Relative Risk; CT: census tract; CI: Confidence Interval. ^‡^ Multivariate Poisson regression model adjusted for age, sex, race, CT median household income and spatial effect (*p* < 0.05). * Seventy-nine cases with a race coded “Other” race were excluded from the analysis.

## Data Availability

Patient data are not publicly available and must be requested from the New Jersey State Cancer Registry upon successful IRB approval.

## Supplementary Materials

The following supporting information can be downloaded at: https://www.mdpi.com/article/10.3390/ijerph20043578/s1, Table S1: Model comparison; Table S2: Distribution and Relative Risk by Race/Ethnicity and CT Poverty Quartile (*n* = 1084 *).
