# Supplementary material for: Disparities in Cutaneous T-Cell Lymphoma Incidence by Race/Ethnicity and Area-Based Socioeconomic Status
Source: Int J Environ Res Public Health. 2023 Feb 17;20(4):3578. doi: 10.3390/ijerph20043578 (PMC9960518; doi:10.3390/ijerph20043578)
Supplement: Supplementary file 1 [file ijerph-20-03578-s001.zip › ijerph-2186233-supplementary.pdf]

Supplemental Materials

Table S1: Model comparison

**Model**	**SIR[1] Min-Max**	**SIR Range[2]**	**GD[3] %**	**DIC[4]**
Age	0.96 - 1.04	0.08	10.35	16602.8
Sex	0.85 - 1.18	0.33	27.32	17365.8
Race	0.87 - 1.17	0.24	18.77	17431.1
CT Median Income	0.86 - 1.17	0.31	18.95	17422.4
CT Poverty	0.88 - 1.14	0.26	20.23	17419.7
Age + Sex	0.92 - 1.06	0.14	14.51	16498.5
Age + Race/Ethnicity	0.96 - 1.06	0.10	12.11	16582.5
Age + CT Median Income	0.93 - 1.05	0.12	12.43	16575.6
Age + CT Poverty	0.94 - 1.06	0.12	14.17	16584.7
Age + Sex + Race/Ethnicity	0.94 - 1.06	0.12	12.29	16520.8
Age + Sex + Race/Ethnicity + CT Median Income	0.93 - 1.07	0.14	12.57	16487.0
Age + Sex + Race/Ethnicity + CT Poverty	0.91 - 1.08	0.17	15.94	16506.1

[1] SIR Standardized Incidence Ratio
[2] SIR Range as difference between min and max SIR
[3] GD Geographic Disparity=Percent remained, unexplained disparities
[4] DIC Deviance Information Criterion

Table S2: Distribution and Relative Risk by Race/Ethnicity and CT Poverty Quartile (N=1084*)

	**CT Poverty Quartile**							
**Race/Ethnicity**	**Very Low (<2.89%)**		**Low (2.89-5.48%)**		**Medium (5.48-10.34%)**		**High (10.34%+)**	
	**N (%)**	**RR‡ (95% CI)**	**N (%)**	**RR‡ (95% CI)**	**N (%)**	**RR‡ (95% CI)**	**N (%)**	**RR‡ (95% CI)**
**Non-Hispanic White**	408 (56)	**1.15 (1.12-3.15)**	216 (29)	**1.06 (1.01-2.88)**	94 (13)	0.98 (0.9-2.67)	15 (2)	Referent
**Non-Hispanic Black**	34 (28)	**1.63 (1.52-5.12)**	46 (28)	**1.66 (1.64-5.26)**	35 (21)	1.09 (0.88-2.96)	51 (31)	**1.49 (1.4-4.44)**
**Hispanic**	23 (18)	1.15 (0.86-3.15)	28 (22)	1.11 (0.86-3.03)	46 (37)	1.12 (0.95-3.05)	28 (22)	0.98 (0.76-2.66)
**Asian/Pacific Islander**	31 (52)	0.96 (0.76-2.61)	18 (30)	1 (0.69-2.73)	9 (15)	0.95 (0.5-2.59)	2 (3)	1.19 (0.17-3.3)

RR: Relative Risk CT: census tract CI: Confidence Interval

‡Multivariate Poisson regression model adjusted for age, sex, race, CT median income and spatial effect ($p<.05$)

* Seventy-nine cases with a race coded "Other" race were excluded from the analysis.
